# The Effect of Phoniatric and Logopedic Rehabilitation on the Voice of Patients with Puberphonia

**DOI:** 10.3390/jcm14155350

**Published:** 2025-07-29

**Authors:** Lidia Nawrocka, Agnieszka Garstecka, Anna Sinkiewicz

**Affiliations:** 1 Department of Otolaryngology, Phoniatrics and Audiology, Ludwik Rydygier Collegium Medicum in Bydgoszcz, Nicolaus Copernicus University in Toruń, Dr. Jan Biziel University Hospital No. 2, Ujejskiego 75 Street, 85-094 Bydgoszcz, Poland; 2 Railway Health Resort Hospital in Ciechocinek, Zdrojowa 17 Street, 87-720 Ciechocinek, Poland

**Keywords:** puberphonia, post-mutational voice instability, mutational falsetto, voice rehabilitation, acoustic analysis

## Abstract

**Background/Objective:** Puberphonia is a voice disorder characterized by the persistence of a high-pitched voice in sexually mature males. In phoniatrics and speech-language pathology, it is also known as post-mutational voice instability, mutational falsetto, persistent fistulous voice, or functional falsetto. The absence of an age-appropriate vocal pitch may adversely affect psychological well-being and hinder personal, social, and occupational functioning. The aim of this study was to evaluate of the impact of phoniatric and logopedic rehabilitation on voice quality in patients with puberphonia. **Methods:** The study included 18 male patients, aged 16 to 34 years, rehabilitated for voice mutation disorders. Phoniatric and logopedic rehabilitation included voice therapy tailored to each subject. A logopedist led exercises aimed at lowering and stabilizing the pitch of the voice and improving its quality. A phoniatrician supervised the therapy, monitoring the condition of the vocal apparatus and providing additional diagnostic and therapeutic recommendations as needed. The duration and intensity of the therapy were adjusted for each patient. Before and after voice rehabilitation, the subjects completed the following questionnaires: the Voice Handicap Index (VHI), the Vocal Tract Discomfort (VTD) scale, and the Voice-Related Quality of Life (V-RQOL). They also underwent an acoustic voice analysis. **Results:** Statistical analysis of the VHI, VTD, and V-RQOL scores, as well as the voice’s acoustic parameters, showed statistically significant differences before and after rehabilitation (*p* < 0.005). **Conclusions:** Phoniatric and logopedic rehabilitation is an effective method of reducing and maintaining a stable, euphonic male voice in patients with functional puberphonia. Effective voice therapy positively impacts selected aspects of psychosocial functioning reported by patients, improves voice-related quality of life, and reduces physical discomfort in the vocal tract.

## 1. Introduction

Voice pitch depends on anatomical conditions, sex, age, the course of puberty, and the completion of the mutational process [1,2]. In boys, sex hormones influence the development and maintenance of male tertiary sexual characteristics, including a low voice [3]. During puberty, the rapid growth of the laryngeal cartilage causes the vocal folds to lengthen and widen. In the midline of the neck, the anterior-superior portion of the thyroid cartilage is accentuated, forming a laryngeal prominence, commonly known as the Adam’s apple (Latin: pomum Adami) [4]. Physiologically, changes in the larynx occur during puberty in boys between the ages of 11 and 16, with the most noticeable manifestation of voice problems—such as hoarseness, voice cracks, and temporary loss of voice—typically occurring between the ages of 13 and 14. These changes are often accompanied by pain in the throat and larynx. In some cases, post-mutational voice problems may persist for many months or even years after the body has reached biological maturity [5].

Puberphonia is a voice disorder that occurs in young men during the post-pubertal period. In phoniatric and logopedic nomenclature, it is referred to as post-mutational voice instability (PMVI) [5], persistent fistulous voice (PFV) [4], mutational falsetto (MF) [6], or functional falsetto (FF) [7]. This condition is defined as the inability of the voice to transition from the high pitch typical of childhood to the lower pitch that is characteristic of sexually mature males, despite the presence of a normal laryngeal structure [8,9,10]. Puberphonia is not a distinct disease entity but a cluster of symptoms, the most prominent of which is an abnormally high-pitched voice that is inappropriate for the individual’s age and gender. This condition is often caused by excessive tension in the neck and laryngeal muscles. Puberphonia usually occurs in isolation and does not lead to additional disturbances in verbal communication [11]. Various therapeutic approaches for treating puberphonia have been described in the literature, including functional methods and, in rare cases, surgical treatment. Voice therapy constitutes the primary intervention, with its effectiveness confirmed in numerous studies [6,10,11]. Varma lists several treatment methods for puberphonia, such as voice therapy, laryngeal manipulation, and laryngeal surgery. However, he emphasizes the key role of voice therapy, which was found to be effective for 11 men who participated in his study [12]. Barmak et al. conducted a retrospective analysis of 31 patients with puberphonia, indicating that voice therapy was an important treatment method [6]. Liang et al. evaluated a group of 26 patients, with an average age of 22.2 years, and demonstrated significant improvement in voice parameters after only four weeks of therapy [7]. De Alwis et al. confirmed the effectiveness of voice therapy in 71 patients aged 14–24 years [11]. In a prospective study, Desai analyzed a group of 30 patients diagnosed with puberphonia and implemented voice therapy as the sole treatment approach. Based on the results obtained, he concluded that voice therapy was effective and recommended it as the primary treatment method for puberphonia [8]. In their study, Dagli et al. presented various therapeutic strategies. Most enrolled patients showed positive results from voice therapy, which significantly reduced their voice pitch. For patients who did not achieve the desired results, the authors considered alternative treatment methods that were unfortunately not described in detail [10]. Manual techniques of repositioning the larynx, which are classified as functional methods, are also worth mentioning. Nelson et al. demonstrated that manual techniques can rapidly enhance voice quality with high clinical efficacy in the early stages of therapy [13]. For cases that do not respond to conservative treatment, surgical procedures such as type III thyroplasty may be used. This procedure involves shortening and reducing the tension of the vocal folds to lower the pitch of the voice. Chowdhury et al. confirmed the effectiveness of this method in six patients aged 16–25 years for whom previous voice therapy was ineffective [14]. Surgical treatment was also described by Kızılay and Fırat, who used it in one patient as an effective alternative in the absence of improvement after functional therapy [15]. Despite the availability of surgical procedures, voice therapy remains the primary and preferred method of treatment for puberphonia, as confirmed by most scientific publications [6,7,11,12].

The etiological criterion distinguishes between puberphonia with organic and functional causes [16]. Endocrine disorders may cause abnormal laryngeal growth. In such a case, diagnosis and treatment by an endocrinologist, along with phoniatric and logopedic voice rehabilitation, are recommended.

In functional puberphonia, psychological factors are indicated as the cause [17,18], particularly emotional immaturity and dependence on significant family members, especially the mother. The absence of a father may also be a contributing factor [19,20]. Puberphonia may negatively impact a man’s mental state [7,21], potentially leading to social and occupational isolation and depression [22]. In some cases, patients require psychotherapeutic support in addition to voice therapy [23]. The main treatment for functional puberphonia is phoniatric and logopedic voice rehabilitation [6], with no need for pharmacotherapy.

Puberphonia is now a more widely recognized voice disorder. Males who experience prolonged voice mutation often decide to consult a phoniatrist, knowing that voice instability can be diagnosed and treated during the period when a low voice, typical of sexually mature males, should appear.

The aim of the study was to evaluate the effect of phoniatric and logopedic rehabilitation on the voice quality of patients with puberphonia. Numerous studies have investigated the efficacy of voice therapy for puberphonia, yet research using standardized self-assessment tools to explore subjective vocal discomfort, voice-related quality of life, and the social and emotional outcomes of voice problems remains limited. The presented analysis expands existing knowledge in this field, providing a multi-faceted evaluation of the effectiveness of voice therapy. It uses three self-assessment tools (VHI, VTD, and V-RQOL), along with acoustic analysis, to evaluate the outcomes of phoniatric and logopedic therapy in males with functional puberphonia.

## 2. Materials and Methods

The study involved 18 males with puberphonia who were undergoing rehabilitation at the Speech Therapy Clinic of the Department of Phoniatrics and Audiology at the Dr. Jan Biziel University Hospital No. 2 in Bydgoszcz, Poland. Ethical approval was obtained from the Ethics Committee of the Collegium Medicum, Nicolaus Copernicus University (KB 644/2018), and all participants provided written informed consent to participate in the study. This prospective study was conducted from October 2018 to December 2023.

The subjects reported an inability to lower their vocal pitch at the age when a mature male voice typically develops.

### 2.1. Phoniatric and Logopedic Rehabilitation Program

The main objective of the rehabilitation process was to produce a stable voice within a frequency range typical of males. In accordance with the accepted phoniatric and logopedic treatment scheme for puberphonia, therapeutic actions were initiated for all patients on the day they were referred to the clinic.

The rehabilitation program was conducted in line with the following plan:

- phoniatric and logopedic examination before voice rehabilitation,
- voice rehabilitation,
- phoniatric and logopedic examination after voice rehabilitation.

ENT, phoniatric, and logopedic examinations were conducted for all subjects prior to and following rehabilitation. The nasal and throat conditions were evaluated, and endoscopic laryngoscopy (EL) was performed. Acoustic analysis was performed on voice samples obtained from recordings of an eight-sentence text in Polish using Diagno Scope Specjalista version 1.3. The samples were collected from nine randomly selected males out of the 18 participating in the study. Parameters such as the mean fundamental frequency (F0) of the speaking voice and formants F1 to F3 were evaluated. Additional acoustic parameters, such as jitter, shimmer, noise-to-harmonic ratio (NHR), relative average perturbation (RAP), and pitch perturbation quotient (PPQ), were assessed in the same group of nine men before and after rehabilitation. The logopedic examination was performed according to the standard logopedic procedure for individuals with functional voice disorders [24]. To assess the effects of phoniatric and logopedic rehabilitation on voice quality, before and after therapy, the subjects completed the VHI, VTD, and V-RQOL self-report questionnaires. The impact of voice disorders on functional, emotional, and physical life domains was assessed using VHI [25] with score ranges of 0–30 (mild voice dysfunction), 31–60 (moderate voice dysfunction), and 61–120 (severe voice dysfunction). Vocal tract disorders were evaluated using the VTD scale, which assesses eight symptoms: burning, tightness, dryness, pain, scratchiness, tenderness, irritation, and a lump-in-the-throat sensation [26]. Each symptom is evaluated using subscales from 0 to 6 that measure frequency and severity. Scores on the frequency scale are interpreted as follows: 0 means ‘never’; a score of 1–3 represents ‘sometimes’; scores of 4–5 indicate ‘often’; and 6 corresponds to ‘always’. On the severity subscale, 0 means ‘no symptoms’; scores of 1–3 reflect ‘mild symptoms’; scores of 4–5 indicate ‘moderate symptoms’; and 6 represents ‘severe symptoms’. The total score can range from 0 to 48 points. The V-RQOL questionnaire, developed by Hogikyan et al. and adapted into Polish by Morawska et al., was used to assess voice-related quality of life [27]. The Polish version contains 10 questions that determine the severity of voice problems and their impact on daily activities. The questions use a scale from 1 to 5, where 1 means ‘not at all a problem’, 2 = ‘to a small extend’, 3 = ‘to a moderate extend’, 4 = ‘to a great extend’, 5 = ‘it could not be worse’. In addition, in accordance with the approach of the authors of the Polish adaptation, an auxiliary Likert scale for self-assessment of voice quality was included, allowing the respondents to choose one of the following answers: poor, average, good, or very good.

Parametric descriptive statistics such as the arithmetic mean, standard deviation, minimum, and maximum were used to describe the collected material statistically. The Wilcoxon signed-rank test was used to analyze the variables VHI, VTD, V-RQOL, fundamental frequency (F0), and formants (F1–F3) before and after voice rehabilitation.

### 2.2. Voice Therapy Protocol

In the initial stage of voice rehabilitation, basic principles of vocal hygiene and emission were introduced. Exercises to improve posture and relax the muscles of the shoulder girdle, neck, and articulatory apparatus were implemented. An important component of this stage was to instruct the patient to breathe correctly using the diaphragmatic-costal breathing method. In breathing exercises, special attention was paid to breathing support and a uniform exhalation phase.

The second stage involved exercises lowering the voice, based on the laryngeal defense reflexes. For this purpose, the voiced cough method and the Boom technique [5,8,28] were used. Vocal exercises based on the vocal-phonetic method [29] were also applied, preceded by the natural laryngeal reflex—a voiced cough. Voice-lowering techniques were tailored to the vocal capabilities of each patient.

The third stage involved exercises designed to maintain a lowered voice. They started with short words, then progressed to sentences, and finally to full texts. The patient gradually adapted to the sound of his lowered voice, learning to recognize and accept it. To preserve the improved timbre and intensity of the voice, exercises were conducted to activate the resonators. During the final stage of voice therapy, emphasis was placed on encouraging the patient to use his lowered voice spontaneously. This was supported by exercises aimed at improving respiratory, phonatory, and articulatory coordination.

Initially, voice rehabilitation sessions were held once a week or every two weeks. Each 40 min session was scheduled according to the patients’ availability. After the voice was lowered, the sessions became less frequent. The same logopedist led the sessions for all participants, ensuring consistency in the therapeutic process. The duration of therapy and the selection of exercises were tailored to each patient’s needs and capabilities. The logopedist determined when to end the therapy based on an assessment of voice stabilization and appropriate pitch. The decision was also based on the patient’s inability to return to the high voice characteristic of the state before therapy. After completing therapy, each patient underwent a phoniatric evaluation to verify the achieved vocal results. The final evaluation considered the patient’s subjective feelings about their voice quality and pitch.

## 3. Results

The study involved 18 male patients aged between 15 and 34 (mean age: 20.5 years). In this group, the duration of voice rehabilitation ranged from 3 to 24 months, with a mean of 8 months and a standard deviation of 5.73. In the perceptual voice assessment before rehabilitation, all subjects presented unstable, high-pitched, soft, age- and gender-inappropriate voices. A shortened expiratory phase and an absence of diaphragmatic–costal breathing were also observed. No additional verbal communication disorders were found. After rehabilitation, a perceptual evaluation showed that the patients’ voices were lower-pitched, resonant, stable, and age- and gender-appropriate. In addition, each patient mastered the technique of diaphragmatic–costal breathing.

### 3.1. Subjective Assessment of Voice Impairment on the VHI Scale

**Figure 1:**
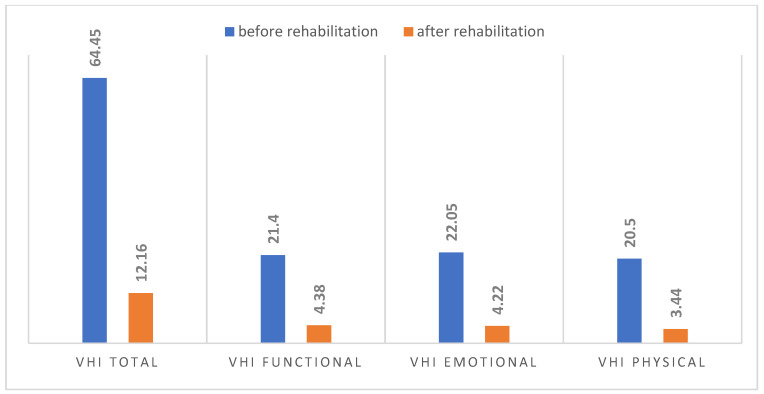
Comparison of VHI scores before and after rehabilitation.

Prior to rehabilitation, the overall VHI score indicated severe voice impairment (64.45), with a standard deviation of 21.27, and a similar degree of voice dysfunction across all scales. To a slight extent, emotional problems prevailed among the respondents (22.05). The voice impairment scores were 21.40 and 20.50 for the functional and physical subscales, respectively. Following voice rehabilitation, the subjects reported a significant improvement in voice quality, with individual scale scores within the normal range. The total score on the VHI scale was 12.16, indicating mild voice impairment, with a standard deviation of 6.98. A comparative analysis of the VHI questionnaire scores before and after rehabilitation is shown in Figure 1.

The differences in self-assessment of voice impairment based on the VHI questionnaire before and after rehabilitation were statistically significant (*p* = 0.00), as shown in Table 1.

### 3.2. Assessment of Vocal Tract Discomfort on the VTD Scale

**Figure 2:**
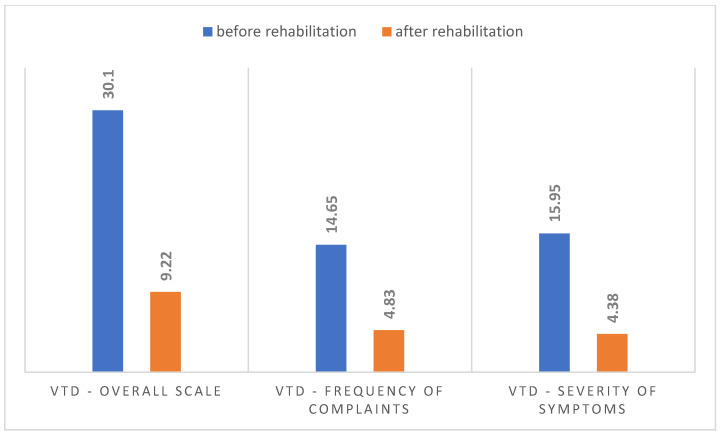
Comparison of VTD scores before and after rehabilitation.

A statistically significant improvement was observed in the overall VTD scale, which assesses discomfort in the vocal tract. Before rehabilitation, the average VTD score was 30.10, while after rehabilitation it decreased to 9.22. The large differences in the results concerned both the frequency of complaints (14.65 before therapy and 4.83 after therapy) and the severity of symptoms (15.95 before therapy and 4.38 after therapy). Figure 2 shows the comparative summary of the VTD questionnaire scores before and after voice rehabilitation.

Significant differences in self-assessment before and after rehabilitation were obtained in both the total VDT scale and its subscales—the coefficient of significance was high (*p* = 0.00), as shown in Table 2.

### 3.3. Assessment of Voice-Related Quality of Life on the V-RQOL Scale

The mean score on the V-RQOL scale before rehabilitation was 43.88 points, while after rehabilitation it increased to 97.93 points. In the assessment of voice quality, according to the Likert scale, where 0 points meant poor quality and 4 meant very good quality, an improvement from 0.25 to 2.55 points was noted. The differences in patients’ scores before and after rehabilitation were statistically significant (*p* = 0.00)—Table 3.

### 3.4. Acoustic Analysis of Subjects’ Voices

Before rehabilitation, the mean fundamental frequency (F0) of the subjects was 200.49 Hz. After therapy, it significantly reduced to a mean of 110.97 Hz. Additionally, after rehabilitation, the maximum value (F0) of the voice decreased from 267.74 Hz to 127.64 Hz, and changes in the values of the first (F1), second (F2), and third (F3) formants were observed. The mean value of F1 was 521.01 Hz prior to rehabilitation and changed to 507.48 Hz afterward. The mean value of F2 was recorded as 1650.67 Hz before therapy and as 1617.53 Hz after. The value of F3 prior to rehabilitation was 2739.88 Hz, and following rehabilitation, it was 2676.23 Hz. The differences in the values of F1, F2, and F3 before and after rehabilitation are shown in Table 4.

Statistically significant differences in the frequency of the laryngeal tone (F0) before and after rehabilitation are shown in Table 5.

Due to the fact that before rehabilitation, the voice frequency of patients with puberphonia was near the mean frequency observed in female voices, normative values for female voices were used to assess voice parameters such as jitter, shimmer, NHR, RAP, and PPQ. After rehabilitation, a decrease in voice frequency was observed, reaching values characteristic of a male voice, and therefore the parameters were assessed based on male voice norms. The results of the acoustic analysis are presented in Table 6.

## 4. Discussion

The absence of a lowered voice in young males may cause problems in their personal, professional, and social functioning [22,23,30]. This study analyzed the effect of phoniatric and logopedic rehabilitation on the voice quality in 18 male patients with PMVI. The ENT and phoniatric assessments revealed no indication for further endocrine diagnostics, as all males were diagnosed with functional puberphonia.

Puberty begins around the age of nine for girls and 11 for boys. This is when androgens, male sex hormones, become active and play a key role in the development of tertiary sexual characteristics, including vocal pitch. During speech, the mean pitch of the adult human voice, which determines the range of sound in which the voice rises and falls by 4 to 8 semitones, is usually within the lower third of the vocal range. For males, this ranges from a to e, and for females, from a to e1 on the musical scale [31]. The mean pitch for sexually mature females is 256 Hz (c1) [31]. In boys, the voice pitch decreased by an average of one octave over a period of 3–6 months in the course of normal voice mutation [32]. The mean pitch of a male voice is 128 Hz (c) [25,31]. In the present study, acoustic analysis before and after rehabilitation showed statistically significant differences in the mean voice pitch score. Prior to therapy, the mean fundamental frequency (F0) was 200.10 Hz, which is similar to that of a typical female voice. After rehabilitation, the voice dropped to a fundamental frequency of 110.97 Hz, characteristic of a male voice. Desai and Prasun obtained results comparable to the results of this study, where after voice rehabilitation of 30 patients with puberphonia, the F0 parameters decreased from 208 Hz to 125 Hz [8]. The effectiveness of voice rehabilitation as a method for treating puberphonia has been confirmed by Barmak, Desai, Varma, and Alam [6,12,33]. In the range of mean fundamental frequency, Barmak showed that in a group of 31 patients with FF, the F0 value was 230.65 Hz before voice therapy and 126.32 Hz after it [6]. Varma achieved an improvement in F0 from 217.45 Hz to 127.50 Hz after therapy [12]. Alam et al. showed that after voice therapy, F0 parameters decreased from 233.85 Hz to 129.28 Hz in 20 patients [33]. The frequency of the laryngeal sound (F0) is the most important parameter of acoustic analysis, indicating the effectiveness or ineffectiveness of voice rehabilitation [22]. In the present study, a 55% reduction in the mean voice pitch score confirmed the effectiveness of the voice therapy used. After rehabilitation, all male subjects had a typical male voice. Also important are the formants, i.e., the resonant frequencies of the vocal tract, that affect the timbre of the voice. In this study, differences were found in the values of F1, F2, and F3 before and after therapy. Prior to rehabilitation, the mean values of F1 and F2 were 521.01 Hz and 1650.67 Hz, respectively, compared to 507.48 Hz and 1617.53 Hz following rehabilitation. The 2.6% change in F1 indicates a slight activation of the posterior (pharyngeal) resonator, which is desirable in the rehabilitation of puberphonia. After therapy, a slight 2% change in F2 was observed in voice projection, which is related to the work of the supraglottic resonating cavities. The difference in F3 results (2.3%), associated with voice intensity before and after rehabilitation, although statistically significant, did not confirm a significant effect of rehabilitation on the function of the resonators. Further research could draw more conclusions through a comparative analysis of F1–F3 values of individual cases before and after voice therapy. In addition, after voice therapy, the intensity of the indicated formants may have changed in patients participating in this study, which was not measurable with the method used but nevertheless had a greater effect on the listener’s auditory perception of voice timbre than the change obtained in the F1–F3 frequencies. This forms the basis for further analyses and the application of adequate methods to investigate differences in the formant intensity associated with resonator activity. After voice rehabilitation, all analyzed acoustic parameters—such as jitter, shimmer, NHR, RAP, and PPQ—improved. The average values of the voice assessment indicators, as determined by comparing the results before and after rehabilitation, were as follows: jitter—0.84; shimmer—0.77; NHR—0.65; RAP—0.76; and PPQ—0.84.

The negative impact of puberphonia on the psychosocial functioning of patients has been confirmed by the results of studies by other authors [7,21,22,23]. The VHI questionnaire is currently a widely used scale for assessing the impact of voice disorders on psychosocial functioning [34,35,36]. The analysis shows that the overall VHI score prior to rehabilitation indicated a high degree of voice dysfunction in all males studied. Although the reported voice dysfunction on the individual scales was similar, the patients scored slightly worse on the emotional subscale. Following completion of the rehabilitation program, the subjects experienced a significant improvement in voice quality, with scores on the individual scales within normal ranges and the overall VHI score indicating mild voice dysfunction. The positive impact of voice therapy on psychosocial functioning is demonstrated by the statistically significant difference in VHI scores before and after rehabilitation. In his study, Alama et al. emphasized the importance of improving the emotional component after rehabilitation, which is essential in the therapy of patients with puberphonia [33].

Post-mutational voice disorders also cause physical symptoms in the laryngeal and pharyngeal regions, so self-assessment of vocal tract problems is important. Before therapy, patients complained about the severity and frequency of pain. After the rehabilitation, these symptoms were significantly reduced, as confirmed by a statistically significant difference in the results obtained from the VTD questionnaire. The results also show the multidimensionality of problems related to PMVI.

Important data on the effectiveness of voice rehabilitation are provided by a comparative analysis of the V-RQOL results. V-RQOL measures the impact of voice disorders on patients’ voice-related quality of life. In this study, patients reported a significantly higher voice-related quality of life after voice rehabilitation than before. The severity of the voice problems was substantially reduced, and voice quality improved, as assessed by the subjects. Due to the lack of reports on V-RQOL scale studies among patients with puberphonia, it was difficult to compare the obtained results with findings from other research.

The mean duration of phoniatric and logopedic voice rehabilitation for the patients studied was 8 months. Daglii et al. performed therapy for 6 months [10]. In his research, De Alwis demonstrated a relatively short period of voice rehabilitation, with a mean duration of 3.5 months [11]. Clinical experience shows that voice pitch can be quickly lowered through implementing appropriate techniques and maneuvers. In the study group, this was most often achieved during the first or second therapy session. The prolonged rehabilitation time and the persistence of a high-pitched voice in the patients were most often due to a specific unconscious inhibition to produce a male-typical voice [5]. Therefore, in the rehabilitation of puberphonia, it is important to positively reinforce any attempt by the patient to use a low voice. In some cases, the inclusion of psychotherapy should be considered. The successful outcomes of voice therapy are largely determined by regular exercises performed by the patient and the speech therapist’s preparation. Many authors agree that voice therapy under the supervision of a qualified speech therapist is an effective method of reducing and maintaining a euphonic voice [6,7,8,10,12,22]. After voice rehabilitation is complete, it is important to evaluate laryngeal function and voice quality. An acoustic analysis by a phoniatrist should also be conducted.

Due to the multifaceted nature of the problems experienced by patients with puberphonia, cooperation between specialists such as a phoniatrist, speech therapist, and psychotherapist is essential in voice rehabilitation.

### Study Limitations

One of the limitations of the study was the acoustic analysis, which was performed only on nine randomly selected participants out of 18. In future studies, acoustic voice assessment should be applied for the entire group in order to obtain a more complete picture of the effectiveness of the therapy and improve the representativeness of the results. Although puberphonia is an increasingly recognized voice disorder, recruiting a representative group of patients for research remains challenging. This may be attributed to the fact that many men do not seek specialist diagnosis or therapy, which is likely driven by a lack of awareness of the problem, limited access to phoniatrists, or an individual approach to voice disorders. As a result, available studies are often based on small, not always fully representative samples, which makes it difficult to generalize the results and requires further, more comprehensive analyses.

The present analysis uses three voice self-assessment tools—VHI, VTD, and V-RQOL—which allow for the assessment of patients’ perception of their voice, including physical complaints and voice-related quality of life. However, these tools only partially address psychosocial functioning and do not provide a comprehensive, structured assessment. Despite this limitation, the study makes an important contribution to the advancement of knowledge about the effectiveness of voice therapy in males with functional puberphonia, also taking into account selected psychosocial aspects reported by patients.

## 5. Conclusions

Phoniatric and logopedic rehabilitation is an effective method of reducing and maintaining a euphonic male voice in functional puberphonia. Effective voice therapy has a positive impact on selected aspects of psychosocial functioning reported by patients, improves voice-related quality of life, and reduces physical discomfort in the vocal tract.

## Figures and Tables

**Table 1:** VHI Questionnaire—basic statistical values before and after voice rehabilitation.

Values of the VHI Questionnaire Before and After Rehabilitation	Wilcoxon Signed-Rank Test, *p* < 0.05			
	*n*	T	Z	*p*
VHI total	18	0.00	3.72	0.00
VHI functional	18	0.00	3.72	0.00
VHI emotional	18	0.00	3.72	0.00
VHI physical	18	0.00	3.72	0.00

**Table 2:** VTD Questionnaire—basic statistical values before and after voice rehabilitation.

VTD Questionnaire Values Before and After Rehabilitation	Wilcoxon Signed-Rank Test, *p* < 0.05			
	*n*	T	Z	*p*
VTD total	18	0.00	3.72	0.00
VTD frequency of complaints	18	0.00	3.62	0.00
VTD severity of symptoms	18	0.00	3.72	0.00

**Table 3:** V-RQOL questionnaire and Likert Scale—basic statistical values before and after voice rehabilitation.

Values of the V-RQOL Questionnaire and Likert Scale Before and After Rehabilitation	Wilcoxon Signed-Rank Test, *p* < 0.05			
	*n*	T	Z	*p*
V-RQOL	18	0.00	3.72	0.00
Likert Scale	18	0.00	3.72	0.00

**Table 4:** Mean values of laryngeal tone and formants (F0–F3) before and after rehabilitation (*n* = 9; total number of subjects = 18).

Laryngeal Tone and Formants	Before Rehabilitation *n* = 9				After Rehabilitation *n* = 9			
	Average Hz	Minimum Hz	Maximum Hz	Standard Deviation	Average Hz	Minimum Hz	Maximum Hz	Standard Deviation
F0	200.49	151.94	267.74	42.00	110.97	90.29	127.64	12.52
F1	521.01	351.45	621.54	82.86	507.48	425.72	594.26	52.51
F2	1650.67	1503.92	1771.82	85.60	1617.53	1448.82	1723.56	88.61
F3	2739.88	2599.16	2885.49	92.87	2676.23	2461.32	2795.20	110.43

**Table 5:** Difference in fundamental frequency (F0) values before and after voice rehabilitation. (*n* = 9; total number of subjects = 18).

Fundamental Tone Frequencies (F0) Before and After Rehabilitation	Wilcoxon Signed-Rank Test, *p* < 0.05			
	*n*	T	Z	*p*
F0—median	9	0.00	2.66	0.00
F0—minimum	9	0.00	2.66	0.00
F0—maximum	9	1.00	2.54	0.01

**Table 6:** Acoustic parameters of voice analysis before and after rehabilitation (*n* = 9) with comparative results.

Acoustic Analysis Parameters	Before Rehabilitation *n* = 9				After Rehabilitation *n* = 9				Results of Comparison Before and After Rehabilitation *n* = 9
	Average	Minimum	Maximum	Standard Deviation	Average	Minimum	Maximum	Standard Deviation	Average
Jitter	14.75	0.11	103.68	21.11	11.50	0.11	68.88	16.15	0.84
Shimmer	8.12	0.50	48.89	30.94	5.88	0.41	30.82	9.61	0.77
NHR	2.60	0.36	10.62	2.12	1.35	0.27	4.68	0.89	0.65
RAP	13.74	0.57	91.21	17.96	10.20	0.52	48.91	10.72	0.76
PPQ	16.65	0.80	89.12	18.33	13.65	0.64	57.97	13.25	0.84

Note: *n* = 9 patients who underwent acoustic voice analysis; a total of 18 people participated in the study.

## Data Availability

All data used to support the findings of this study are available from the corresponding author upon request.

## Abbreviations

The following abbreviations are used in this manuscript:

PMVI	Post-mutationalvoiceinstability
MF	Mutationalfalsetto
PFV	Persistent fistulous voice
VTD	Vocal tract discomfort
VHI	Voice Handicap Index
FF	Functional falsetto
V-RQOL	Voice-RelatedQuality of Life
